# Editorial: Immunotherapy in acute myeloid leukemia (AML) and acute lymphoblastic leukemia (ALL)

**DOI:** 10.3389/fimmu.2024.1501760

**Published:** 2024-10-17

**Authors:** Omer Jamy, Fuling Zhou, Joshua Zeidner

**Affiliations:** ^1^ Department of Medicine, University of Alabama at Birmingham, Birmingham, AL, United States; ^2^ Department of Hematology, Wuhan University, Wuhan, China; ^3^ Department of Medicine, University of North Carolina at Chapel Hill, Chapel Hill, NC, United States

**Keywords:** acute lymphoblastic leukemia, acute myeloid leukemia, immunotherapy, acute leukemia, translational research

Harnessing the immune system to treat cancer was first described by Dr. William Coley in the 1890’s after he developed a Streptococcal toxin to treat metastatic Sarcoma patients (1). Dr. Coley’s omniscience, however, did not translate clinically until almost a century later whereby immunotherapy approaches have led to a paradigm shift in the management of many solid tumors and hematologic malignancies. Allogeneic stem cell transplantation (alloSCT) has offered the ability to cure patients with high-risk hematologic malignancies via a known immunologic-based graft-versus-leukemia effect. Other immunotherapy approaches for hematologic malignancies have mainly been limited to B-cell neoplasms with bispecific T-cell engagers (BiTEs) for acute lymphoblastic leukemia (ALL), chimeric antigen receptor (CAR) T cells for ALL and lymphoma and immune checkpoint inhibitors (ICPIs) for lymphoma (2).

In the past decade, we have made significant progress in understanding the biology of acute myeloid leukemia (AML) and ALL, including studying the underlying immune systems at play (3). This has led to the investigation of innovative T-cell-based treatment strategies for these diseases. Within this context, we created this Research Topic to welcome studies on further understanding the immune landscape of AML and ALL, along with both pre-clinical and clinical data of immunotherapy in these settings and its implication in the real-world.

## AML

A breakthrough in the treatment of AML has been the approval of venetoclax (VEN), a BCL-2 inhibitor. The combination of VEN and a hypomethylating (HMA) is now standard of care for older and/or frail patients with AML and several VEN-based combinations are under investigation (4). Corradi et al. reported on the immune repertoire of 27 AML patients treated with VEN/HMA and provided insight into VEN-specific modulation of the immune cell compartment. PD-1^+^TIM-3^+^ CD4^+^ and CD8^+^ T-cells have been associated with poor outcomes in AML and the authors demonstrated a VEN-dependent decrease in this cell population, highlighting a potentially beneficial effect of VEN on functional T-cell response. A VEN-specific increase in TIM-3 expression on CD8+ T-cells was also observed, supporting the ongoing trials of TIM-3 inhibitors plus VEN (5). Lastly, a VEN-dependent decrease in cytokine-secreting non-suppressive Tregs was demonstrated but the significance of this finding needs further exploration. These results help us understand the impact of BCL-2 inhibition on the immune microenvironment and give way to future research into resistance mechanisms.

The next two articles focused on refining AML risk stratification. Cao et al. investigated the significance of nicotinamide adenine dinucleotide (NAD) metabolism-related genes and identified eight genes with prognostic relevance. A multivariable model termed the 8-gene NAD metabolism score (NADM8) was developed to stratify patients in high and low-risk groups and validated in independent AML cohorts. Interestingly, the immune microenvironment of NADM8 high-risk patients exhibited a higher expression of immune checkpoint markers relative to low-risk patients. Since AML is thought to reside in an immunosuppressive state, the NADM8 signature may identify patients with a unique immune state potentially responsive to immunotherapy. Li et al. developed and validated a CD8+ T-cell-related immune risk score by identifying a 15-gene prognostic signature using regression on the genes expressed between CD8+ T-cell high and low-groups. Incorporation of the model into the European LeukemiaNet (ELN) 2022 risk stratification model resulted in improved outcomes prediction. These studies highlight that the immune microenvironment in AML is associated with prognosis and may serve as a ‘biomaker’ for therapeutic intervention.

Lastly, Wang et al. provide a clinical report of 15 patients with relapse/refectory (r/r) AML treated with tislelizumab (anti-PD-1) plus conventional chemotherapy. They specifically focus on outcomes after alloSCT given the concern for increased risk of graft vs. host disease (GVHD) in patients receiving pre-transplant immunotherapy. The combination did not result in an exacerbated incidence of severe GVHD. Survival outcomes were comparable to previous reports, but these findings need to be validated in larger cohorts.

## ALL

The treatment landscape of r/r B-cell ALL has been reshaped by the approval of blinatumomab, inotuzumab ozogamicin and CAR-T therapy, ushering in an exciting era of immunotherapy and setting up the new goal of moving towards an era without cytotoxic chemotherapy. Poveda-Garavito et al. provide a comprehensive review on the role of the tumor immune microenvironment (TIME) in the diagnosis and progression of B-cell ALL. They explore the interaction between TIME and leukemia to understand pathways of resistance and identify strategies to manipulate TIME to facilitate antitumor immunity. These insights into the microenvironment of B-cell ALL will hopefully translate into more effective and novel therapies for this disease. Moving to the clinical side, Johns et al. report on two cases of pre-CAR-T sub-clinical non-CNS extramedullary (EM) relapse after novel targeted therapy for r/r B-cell ALL and add to the growing body of literature the significance of identifying relapse patterns in the era of immunotherapy along with the role of imaging studies for EM relapse.

T-cell ALL remains an aggressive disease to treat due to complicated biology coupled with lack of effective therapeutics (6). Ren et al. provide insights into the tumor microenvironment of T-ALL and highlight the functional abnormalities in T-cells, NK cells, and immunological checkpoints along with impaired inflammatory markers observed in this disease. They associate decreased expression of GZMA, IL7R, GZMK, CCL5, CCR7, PRF1, TIGIT, CTLA4, KLRB1, and KLRD1 genes with inadequate long-term clinical immunological response in T-ALL. Clinically speaking, whereas CAR-T therapy has come a long way in B-cell ALL, we are noticing its efficacy in T-cell as well. Song et al. provide an update on two cases of pediatric r/r T-cell ALL cases achieving remission with donor CD7 CAR-T therapy and proceeding to alloSCT with excellent long-term outcomes. These results are encouraging but ongoing studies of CD7 CAR-T therapy are needed to inform us further.

In conclusion, our topic highlights the keen interest of exploring the tumor immune microenvironment, in AML and ALL, to better understand disease biology, risk stratification and response to therapy. We also observe that these findings are being translated into clinical studies to treat challenging entities such as r/r AML and T-cell ALL, indicating the promising potential of immunotherapy in leukemia harking back to the clairvoyant observations by Dr. Coley over 130 years ago.
